# Supplementary material for: Are carbon emissions trading and green financial instruments synergistic? -Comprehensive quantitative research based on content analysis
Source: PLoS One. 2024 Mar 7;19(3):e0298601. doi: 10.1371/journal.pone.0298601 (PMC10919601; doi:10.1371/journal.pone.0298601)
Supplement: S1 Data — (ZIP) [file pone.0298601.s001.zip › Data/地级市Z_F工作报告.zip!/─■╧─/╩»╫∞╔╜╩╨/└┤╫╘╣1⁄2╓┌║┼í╛┬φ┐╦╩2╛▌═°í┐_2017.docx]

各位代表：

现在，我代表市人民政府向大会作政府工作报告，请予审议，并请市政协委员和其他列席人员提出意见。

一、过去四年工作回顾侯卫东官场笔记

市十三届人大一次会议以来，面对外部环境复杂多变、经济下行压力加大的严峻形势，市人民政府认真贯彻落实自治区党委、政府和市委的决策部署，团结带领全市广大干部群众，主动适应经济发展新常态，砥砺奋进，攻坚克难，坚定不移推进产业民生生态转型发展，建设开放富裕和谐美丽新型工业城市，全市经济社会各项事业健康发展，为全面建成小康社会奠定了坚实基础。

——坚持稳中求进，综合实力迈上新台阶。始终把发展作为第一要务，准确把握新常态下经济发展的客观规律，在稳增长和调结构的平衡点上精准发力，全力促进经济在合理区间运行。预计到2016年底，实现地区生产总值505亿元，同比增长7%，年均增长8.4%；完成固定资产投资500亿元，同比增长0.2%，年均增长7%；城乡居民人均可支配收入分别达到25860元和11820元，同比增长7%和7.5%，年均增长8.1%和9.3%；累计完成一般公共预算收入111亿元。四年来，共争取到全国循环经济示范城市、全国小微企业创业创新基地示范城市等16项国家级示范试点政策，获得国家和自治区各类支持资金299亿元，有力支撑了转型发展。在全国67个资源枯竭型城市转型绩效考核中，从2013年的第52名跃升至2015年的第16名，三年前进三大步，从达标跨入良好等次，居西部同类城市前列。

——坚持转型升级，质量效益得到新提升。制定实施产业转型升级和结构调整“1+3”方案、老工业基地调整改造规划，加快产业转型升级。工业四大优势产业集群和四个特色产业初步形成，“4+4”产业增加值比重达到46%。实施石嘴山经开区、生态经济开发区51个国家循环化改造项目，处置“僵尸企业”90多家，累计淘汰落后产能351万吨，单位GDP能耗下降14.6%；大地循环、晟晏实业、嘉峰化工等一批企业通过技改升级、兼并重组做大做强，焕发新的生机和活力；恒达纺织、丽珠药业、中利线缆等一批调结构的新项目、大项目建成投产。现代物流和文化旅游业加快发展，中阿物流园、石嘴山保税物流中心（B型）启动建设，开通了“陆港号”集装箱专列；奇石山文化旅游区、煤城·记忆等项目有序推进。

汉唐九街、惠农东大街商圈、平罗国际建材城等建成投运，电子商务、健康养老等产业蓬勃发展，服务业比重提高3个百分点。大力推动农业“一优五特”产业提质增效，优势特色产业占农业比重达到86%，国家农业科技园区、河东现代农业示范区加快建设。实施百万亩盐碱地改良、国土整治等工程，农业发展基础不断夯实，粮食生产实现“十三连增”。

——坚持民生优先，社会事业取得新进步。在财力十分困难的情况下，每年办理一批民生实事，累计投入民生领域资金240亿元，占一般公共预算支出的74%。大力发展教育事业，在全国率先实现义务教育基本均衡发展。深化医药卫生体制改革，公立医院实行药品零差价，基本公共卫生服务水平明显提升。建设星海中学、儿童医院、妇幼保健院等一批重点项目，不断改善教学和医疗条件。深入推进文化惠民工程，整合57项文化体育资源免费向群众开放，成功创建国家公共文化服务体系示范区。连续举办国际铁人三项赛、国际标准舞公开赛等活动，丰富了群众文化体育生活。健全就业服务体系，提高就业质量，全市新增就业8万人。在全区率先实行居民医疗、养老、大病保险统筹，社会保险受益人群不断扩大。建成了市敬老院、老年活动中心、失能失智护理院等养老服务机构。投入各类资金100多亿元，建设棚户区安置住房和保障性住房4.2万套，从根本上解决了12万困难群众住房问题。搬迁安置移民4.5万人，推进“四项脱贫计划”和“五项助力行动”，对建档立卡贫困人口实施精准脱贫。加强食品药品安全监管，完成餐饮服务单位“明厨亮灶”改造1841家。社会治理、平安建设、信访规范化等工作走在全区前列，荣获全国“六五”普法先进城市、全国残疾人工作先进单位、全国创建幸福家庭活动示范市。妇女、儿童、老龄事业长足发展，民族宗教、防震减灾、统计调查、粮食供销、电力通讯、金融保险等工作不断加强。

——坚持统筹推进，城乡面貌发生新变化。扎实开展以创建全国文明城市为龙头的“六城联创”，成功创建国家森林城市、国家园林城市和全国双拥模范城市，荣获全国绿化模范城市荣誉称号。完善城乡规划，大力推进城乡建设，新建住宅小区90个，改造老旧住宅小区173万平方米，建设美丽小城镇9个、美丽乡村47个，城乡一体化明显加快，常住人口城镇化率由71%提高到74%。实施了城市亮化工程，新建市民休闲森林公园3个，城市形象大为改观。建成石银同城化干道、简滨公路、石嘴山黄河公路大桥等一批重大基础设施项目。建设港湾式公交站亭110个，新增公交线路6条、新能源公交车110辆，建成大武口区慢行交通系统，群众出行更加便利。实施了贺兰山东麓生态防护林、采煤沉陷区治理、星海湖湿地恢复整治等重点生态工程，全市森林覆盖率达到12.5%，城市建成区绿化覆盖率达到40%。强力推进大气、水体和土壤污染治理，规范整治煤炭市场，扎实整改中央环保督察组反馈问题，环境质量不断改善。

——坚持创新驱动，经济发展增加新动能。加快推进科技创新，深入实施中科协创新驱动助力工程，全市100多项科技成果达到自治区先进水平，培育高新技术企业和科技型中小企业81家，建成稀有金属特种材料国家重点实验室，建设国家和自治区级科技创新平台16个，万人发明专利拥有量达到3件，科技对经济增长的贡献率达到48.6%，石嘴山高新区升级为国家级开发区。推动大众创业、万众创新，建成小微企业创业创新基地（孵化园）31个，成功举办全市首届创业创新大赛，高标准建设宁夏高技能人才培训基地，被列为全国职业培训示范城市。创新金融服务方式，成立鑫鼎担保公司，设立了产业引导基金和四个子基金；组建债权人委员会，政银企联动，推动金融风险有效化解；推进直接融资，17家企业在新三板、天交所和上海股交中心挂牌，4家企业发行债券融资52亿元，助推了产业转型升级和结构调整。

——坚持破立并举，改革开放呈现新局面。政府机构改革全面完成，行政审批制度改革不断深化，制定公布了政府部门权力清单、责任清单，市本级行政审批事项减少35.6%，非行政许可审批事项全部清零。建成启用新的政务服务中心，做到审批部门全进驻、审批事项全覆盖、公共资源交易全进场。推进商事制度改革,实行“三证合一、一照一码”登记制度，全市小微企业达到10844家、个体工商户达到40299家，分别是2012年底的4.3倍、2.1倍。全力推进农村改革，实施了多项国家级农村改革试点，土地经营管理制度改革成效显著，经验在全国、全区交流。市属国有企业、招投标、户籍制度等改革取得积极进展。以开放促发展，实施了内陆开放型经济试验区建设十个重大专项，与14个国内外友好城市开展广泛交流合作。招商引资成效明显，四年累计引进招商项目1015个，到位资金1383亿元，年均增长12.1%。

——坚持转变职能，自身建设得到新加强。健全政府决策规则和程序，完善政府法律顾问制度，推进依法行政，政府决策的科学化、规范化水平明显提高。坚持向市人大及其常委会报告工作，向市政协通报情况，办理人大代表建议246件、政协提案728件。强化政府部门日常监督，实行按季考评，建立督查评估推进机制，有效促进了工作落实。畅通社会监督渠道，加大政务公开力度，开展电视问政，设立政务微博，发挥人民议政网作用，推动解决了一批热点、难点问题。加大对重点领域、重大项目的审计监督，确保各类资金合理使用。严肃责任追究，行政问责单位46个、干部85人。认真落实中央“八项规定”精神，扎实开展群众路线教育实践活动、“三严三实”专题教育、“两学一做”学习教育，持续整治“四风”，严肃查处并公开通报了45起不作为、慢作为、乱作为典型案例，政风行风明显好转。

各位代表，四年来，我们战胜了诸多困难，经受了严峻考验，取得的成绩来之不易，这是自治区党委、政府和市委坚强领导的结果，是市人大、政协大力支持的结果，是全市人民团结一心、不懈奋斗的结果。在此，我代表市人民政府，向辛勤工作在各条战线的广大干部群众，向人大代表、政协委员，向各民主党派、工商联、人民团体和各界人士，向驻石中央和区属单位、驻石部队、武警官兵、公安干警，表示衷心的感谢和崇高的敬意！

在总结成绩的同时，我们更加清醒地认识到，全市经济社会发展还面临许多困难和问题，主要是：经济下行压力依然较大，部分企业生产经营困难，固定资产投资增长乏力，财政收支矛盾日益突出；经济结构不尽合理，产业层次仍然较低，资源环境约束趋紧；开发区产业布局不合理、基础设施不完善、环境形象亟待改善，城市吸引力、辐射能力亟需加强；基本公共服务供给质量不高，城乡居民收入增速放缓，脱贫解困和改善民生的任务艰巨；对标全面小康社会指标体系，文化及相关产业占GDP比重、单位GDP能耗、主要污染物排放强度等9个方面指标实现程度不到60%，短板十分突出。与全区其他地市相比，我市的竞争优势有所减弱，面对前有标兵、后有追兵的严峻形势，稍有松劲懈怠，就会掉队落伍。我们必须本着对人民高度负责的精神，坚持目标导向与问题导向相结合，在破解难题上出实招，在补齐短板上用实功，精准施策，持续用力，努力解决好发展中的困难和问题。

二、今后五年主要任务二号首长

今后五年，是全面建成小康社会的决胜期。刚刚闭幕的市第十次党代会，准确把握形势，全面审视市情，科学绘制了转型发展走新路、全面振兴创新业、决胜全面小康的宏伟蓝图，这是全市人民的热切期盼和共同愿望。落实党代会精神是政府的政治责任，我们要振奋精神、凝聚力量，开拓创新、真抓实干，奋力将宏伟蓝图变为现实，谱写石嘴山发展的新篇章。

按照市第十次党代会的总体部署，政府工作的总体要求是：高举中国特色社会主义伟大旗帜，坚持以邓小平理论、“三个代表”重要思想、科学发展观为指导，全面贯彻党的十八大和十八届三中、四中、五中、六中全会精神，深入贯彻习近平总书记系列重要讲话精神和治国理政新理念新思想新战略，按照“五位一体”总体布局和“四个全面”战略布局要求，以新发展理念为统领，主动适应经济发展新常态，坚持发展第一要务，坚持民生为本，以提高发展质量和效益为中心，紧紧围绕“四个宁夏”建设，大力实施创新驱动、协调联动、绿色发展、开放带动、共建共享五大战略，打造石嘴山经济升级版，打响“山水园林·尚工逸城”城市品牌，转型发展走新路，全面振兴创新业，持之以恒推进产业民生生态转型，建设开放富裕和谐美丽石嘴山，努力实现经济繁荣、民族团结、环境优美、人民富裕，为决胜全面小康而不懈奋斗！

主要奋斗目标是：到2021年，地区生产总值年均增长7%左右，固定资产投资年均增长10%左右，一般公共预算收入与经济增长同步，城乡居民人均可支配收入年均分别增长7.5%和8.5%。

今后五年，我们要围绕优势再造和弯道超车，打造石嘴山经济升级版；聚力提升品质和补齐短板，打响“山水园林·尚工逸城”城市品牌，落实“十三五”规划及专项规划，抓好以下重点工作：

（一）着力推进产业转型，全面提升竞争优势。全力以赴打好产业转型升级和结构调整攻坚战，构建特色鲜明的现代产业体系，再造产业发展新优势，不断提升区域经济竞争力。

推动工业迈向中高端。坚持做大总量和调优结构并重、改造提升传统产业和培育新兴产业并举，持之以恒实施升级改造、增量培育、龙头带动和开发区提档升级“四大工程”，促进工业发展向质量效益型转变。发展壮大产业集群。落实中国制造2025宁夏行动纲要，推动装备制造、新材料、电石化工、冶金四大优势产业集群向创新驱动、绿色低碳、智能制造发展转型，积极发展储能材料、晶体材料、特高压配电设备、高端铸造等新产业和新产品，加快建设国家新材料生产研发基地、现代装备制造产业基地、特色冶金产业基地、世界氰胺之都，打造两个产值过200亿元、两个产值过100亿元的产业集群。大力发展新能源、生物医药、新型煤化工、现代纺织四个特色产业，形成一批先导性、带动性强的新兴产业。到2021年，新兴产业占GDP比重达到50%以上，非煤产业占工业总产值比重达到70%以上。做大做强龙头企业。组织企业开展行业对标，推进以智能化、绿色化、服务化为重点的技术改造，每年实施一批重点企业技术改造项目，提高技术改造投资占工业投资比重。继续支持骨干企业通过兼并重组、技术改造不断壮大规模实力，提高产业集中度，培育2—3家销售收入过百亿元、3—5家销售收入过50亿元的大企业、大集团。发扬“工匠精神”，加强品牌建设，聚焦长项，培育一批“专、精、特、新”中小企业。促进开发区提档升级。严格落实开发区总体规划，按照功能定位合理布局产业和项目，推动开发区差异化、特色化发展。加快现代纺织园、医药产业园、电子材料产业园、轻质材料产业园等专业园、园中园建设，提高产业承载能力。创新开发区管理体制，赋予相应的管理权限，增强服务功能。加强开发区道路、供热、供气、供排水、环保等基础设施建设，推进环境综合整治，打造低成本园区，为企业提供良好的发展环境。力争形成3个产值过300亿元的开发区，全市四个开发区工业总产值突破1000亿元。

提速发展现代服务业。把现代服务业作为实现弯道超车、走新路、创新业的重要途径，大力培育引进新产业、新业态、新模式，推动服务业“发展提速、比重提高、结构优化、质态提升”，服务业增加值占GDP比重达到36%以上。实施现代物流提升工程。加快中阿物流园、石嘴山保税物流中心（B型）建设，加强与天津港、曹妃甸港和乌力吉口岸合作，积极培育综合型、专业型物流龙头企业，提升石嘴山作为宁北及蒙西地区物流中心的辐射带动作用，逐步形成区域大宗商品交易中心。实施全域旅游开发工程。推进大沙湖旅游区建设，提升北武当景区、奇石山文化旅游区、煤城·记忆等景区品质，加强旅游线路规划和策划营销，打响“观景沙湖·休闲星海”旅游品牌；引进旅游龙头企业，抓好27处工业遗产的保护和开发，形成新的景点；加快自驾车营地、旅游集散中心等配套设施建设，全面提高旅游交通服务水平和游客接待能力，把一县两区文化旅游的特色亮点聚点成线、连线成面，推动单一景点旅游向全域旅游转变。实施电子商务推进工程。创建电子商务示范基地，每个县区抓好1—2个规模大、引领作用强的电子商务产业园；支持本地电商与知名电商平台开展合作，引进培育一批有影响力的电商企业；大力发展农村电子商务，推进农村电商“村村通”；依托外贸进出口企业，积极发展跨境电子商务。实施科技金融培育工程。高度重视金融业发展，加快培育和发展互联网金融等新业态，力争引进科技金融机构10家、上下游相关企业30家，建成宁夏乃至西北重要的科技金融产业聚集地，创建科技金融应用示范城市。实施健康养老服务工程。大力发展健康养老等生活性服务业，依托星海湖、红柳湾、天河湾生态环境优势，鼓励社会资本参与发展健康养老产业，满足日益增长的健康养老需求。鼓励传统商贸企业创新经营模式，推进老城区商业网点升级换代和功能更新，加快特色商业街区建设，不断引进知名商贸企业，活跃消费市场，促进服务业优化升级。

大力推进农业现代化。坚持用工业化理念发展现代农业，推动一二三产业融合发展，促进农业增效、农民增收。实施农业产业化提升工程。抓好国家农业科技园区“一园四区”及河东现代农业示范区建设，加快新品种、新技术、新机械推广应用，新培育农业经营主体100家，推动农村土地向专业大户、家庭农场和农村经营合作组织流转，发展多种形式的适度规模经营。大力培育农业产业化龙头企业，实施一批重大项目，培育一批知名品牌，依托中粮米业、宁羊牧业、华泰农瓜菜、泰金种业等企业，建设在宁夏乃至全国有影响的优质农产品精深加工基地、水产品基地、农作物制种基地、清真牛羊肉生产基地和优质奶源基地，促进“一优五特”产业做精做特，带动农民持续增收。实施耕地质量提升工程。加强耕地保护和土壤改良，加大农业基础设施建设投入力度，推进农业综合开发、盐碱地改良、中低产田改造和高标准农田建设，提升农业综合生产能力和质量效益，夯实现代农业发展基础。

（二）着力推进创新驱动，全面培育发展动能。深入实施创新驱动战略，创建国家创新型城市，使创新成为引领发展、弯道超车的强大动力。

提升科技创新能力。强化企业主体地位，引导企业建设技术创新中心、院士专家工作站、科技成果转化服务器等科技创新平台，支持科研院所在我市建立分支机构。聚焦有色金属新材料、高端装备制造、锂电池材料等产业，鼓励企业与科研机构、高校开展产学研合作，加快科技成果转化应用。深入实施科技创新工程、科技育龙工程，突破一批制约转型发展的关键技术，着力培育一批科技型企业。健全完善科技创新投入机制，落实企业科技创新后补助等政策，引导企业和社会增加科技创新投入。到2021年，全市高新技术企业达到20家，R&D支出占GDP的比重达到2%以上，万人发明专利拥有量达到4件，高新技术产业增加值占工业增加值的比重达到20%以上，科技进步贡献率达到55%，以科技创新提升转型发展动力。

全力推进创业创新。加快全国小微企业创业创新基地示范城市建设，提升各开发区创业孵化园、大学生创业孵化园、残疾人就业创业孵化园服务功能，建设宁夏智能硬件产业园、石嘴山启明星众创空间、宁夏理工学院大学生创业创新体验中心等创业基地、创新平台，努力打造西北地区乃至全国创业创新亮点。学习借鉴众创、众包、众扶、众筹等新模式，促进产需对接、技术共享、产业融合。完善创业创新扶持机制，鼓励支持农民工、大学生、退役士兵等返乡创业。大力优化发展环境，营造重商、扶商的浓厚氛围，激发非公有制经济活力和创造力。

发挥人才创新作用。突出“高精尖缺”导向，采取走出去、引进来相结合的方式，不求所有、但求所用，大力培养和引进高层次和技能型人才，尤其注重引进现代企业管理人才、金融人才，为转型发展提供智力支持。精准对接产业发展需求，依托宁夏理工学院、宁夏工业学校，打造一批具有石嘴山特色、西部领先的品牌专业，每年培养各类技能人才4000人以上。加强企业家队伍建设，大力推行校企合作，鼓励企业在一线培养人才。健全人才评价激励机制和多元化投入机制，完善和落实人才政策，实施“名师”“名医”工程，让各类人才创业有机会，干事有舞台。

（三）着力推进改革开放，全面增强发展活力。坚持改革不停顿、开放不止步，把激活内力与借助外力更好地结合起来，全力打造内陆开放新高地，为转型发展、全面振兴注入新的活力。

推进供给侧结构性改革。深入推进“三去一降一补”，差别化地去产能，推动煤炭、电石、铁合金等产业整合和兼并重组，坚决淘汰落后产能，有序处置“僵尸企业”。多措并举去库存，采取棚户区改造货币化安置、鼓励农民进城购买住房等措施，有效化解房地产库存，促进房地产业健康发展。积极稳妥去杠杆，推动多层次资本市场建设，鼓励企业通过上市、发债等渠道直接融资，优化融资结构；规范各类融资行为，发挥债权人委员会作用，有效防范和化解金融风险。千方百计降成本，落实国家税费减免政策，推动企业降本增效，增强竞争优势。坚持不懈补短板，着力解决科技投入不足、基础设施薄弱等问题，增强发展的全面性、协调性、可持续性。

深化重点领域改革。深化“放管服”改革，实行政府部门权力清单、责任清单动态管理，进一步精简和下放审批事项；大力推行“互联网+政务服务”，健全完善项目并联审批和网上审批制度，不断提高审批效率和服务水平；全面实施“五证合一、一照一码”，推行“双随机一公开”监管，营造创业创新良好环境。深化农村综合改革，坚持试点引领，大力推进“6+7”农村改革，推进集体所有权、农户承包权和土地经营权“三权分置”，推进农村土地征收、集体经营性建设用地入市、宅基地制度改革。深化户籍制度改革，全面放开落户限制，探索建立来我市落户高校毕业生在市内购房补贴制度。深化市属国有企业改革，完善现代企业制度，发展混合所有制经济。深化招投标制度改革，推进公共资源交易“一网三平台”建设，进一步规范交易行为，净化交易市场环境。深入推进投融资体制改革，组建国有资本运营公司，推广运用PPP模式，撬动引导更多社会资本来我市投资。

大力开展经济合作。主动融入“一带一路”，借助中阿博览会开放平台，加强与阿拉伯国家和穆斯林地区、国内外友好城市的交流，提升经贸、技术、人才等方面的合作水平。支持企业抱团“走出去”，开拓境外市场，发展对外贸易。鼓励企业参与中沙工业园和阿曼杜古姆产业园建设，提高经济外向度水平。积极对接京津冀、长三角、珠三角等发达地区，承接东中部地区产业转移，支持有条件的开发区共建产业转移园区。参与呼包银榆经济区建设，推动与乌海、阿拉善、银川等毗邻地区基础设施有效衔接，强化现代物流、生态环保、文化旅游等方面的务实合作。加大精准招商力度，加强与国内著名商会、行业协会和专业展会的对接，办好区域性招商活动，推进产业链招商、以商招商、以企招商，紧盯央企国企、行业龙头企业，提高招商引资项目质量和水平，力争在招大商、引大资上有新突破。继续办好大型文化体育活动，打造特色节会品牌，提高对外知名度。

扩大对外开放通道。打造铁路枢纽，向东畅通与天津港的物流通道，向西推动“宁新欧”铁路班列延伸至惠农陆路口岸，向南连通内陆发达地区，向北推动石嘴山—乌力吉口岸连接线建设，积极推进包头至银川高铁石嘴山段建设。融入全区“一港三支线”空中交通格局，建设石嘴山沙湖机场，扩大交通辐射半径。完善公路网络，实施乌玛高速石嘴山段等一批重大项目，推动城市出口与过境干线互联互通，构建便捷、开放、高效的交通体系。

（四）着力推进城乡建设，全面打造宜居家园。加快推进以人为核心的新型城镇化，扩大“山水园林·尚工逸城”城市品牌影响，全面提升城市品质和竞争力，让城市更加宜居、城镇各具特色。

完善城市综合功能。落实《宁夏空间发展战略规划》，推进多规合一，严格空间规划管控，做到一本规划管全局、一张蓝图绘到底。优化城市功能布局，推动中心城区差异化发展。统筹城市地下和地上、旧城和新城、城区和开发区基础设施建设，实施地下综合管廊、雨污分流、污水厂提标改造、垃圾无害化处理等工程，合理布局建设停车场、市民公园和免费公厕，提升城市综合承载能力。推进城市公交国有化及城乡公交一体化，方便市民出行。推动城市管理重心下移，加强交通秩序、环境卫生、公共场所、小区物业管理，推进智慧交通、智慧城管、智慧社区建设，提高城市治理和服务水平。

提升城市品质形象。坚持不懈推进以全国文明城市为龙头的“六城联创”，力争成功创建国家卫生城市、全国社会治安综合治理优秀城市和全国文明城市，增强城市吸引力和辐射力。开展城市设计，注重对城市建筑景观与色彩、标志性建筑等要素的建设引导，塑造城市特色风貌。继续实施城市亮化工程。有序推进城市更新改造，2020年前基本消除棚户区、城中村和城市危房。深入挖掘“工矿文化”“移民文化”等珍贵文化资源，留住记忆、感触历史，培育新型工业城市文化，提高市民的归属感和认同感。以优质的教育、医疗、养老服务，聚人气、增活力，建设宜居城市，留住本地人口，吸引更多外地人口来石创业就业、生活居住。

统筹城乡一体发展。完善农民进城落户配套政策，鼓励农民进城居住、就业，提高户籍人口城镇化率。促进乡镇差异化发展，规划建设历史文化名镇名村，推进星海、红果子、陶乐、黄渠桥等特色小镇建设。优化村庄规划布局，采取适当集中居住方式，逐步消除“空心村”。推动基础设施和公共服务向农村延伸，因地制宜抓好农村垃圾、污水集中处理和改水改厕，持续改善乡容村貌，加快建设田园美、村庄美、生活美、风尚美的美丽乡村。

（五）着力推进生态文明，全面改善环境质量。牢固树立“绿水青山就是金山银山”的理念，以国家生态文明先行示范区建设为统领，推动形成绿色生产生活方式，加快改善生态环境。

大力开展生态建设。持之以恒做好造林绿化、城市美化、水系优化“三篇文章”，巩固国家森林城市、国家园林城市创建成果，开展国家生态城市创建工作。推进大规模造林绿化，实施绿色通道建设、城市绿地提档升级等重点生态项目，构建贺兰山东麓生态防护带、中部平原绿洲景观带、东部防沙治沙带“三大生态安全屏障”。到2021年，全市森林覆盖率、城市建成区绿化覆盖率分别达到13.5%和41%。加快推进水生态文明城市建设，保护重点湖泊湿地生态环境，实施沙湖、星海湖、镇朔湖等水系连通工程，优化水系，改善水质。

切实加强环境保护。深入实施“蓝天碧水·绿色城乡”专项行动，集中解决环境污染问题，持续改善环境质量。开展重点行业清洁化改造，推进燃煤电厂、燃煤锅炉、工业窑炉、施工扬尘和机动车尾气污染治理，空气质量优良天数达到78%以上。强化饮用水水源地保护，加大市域内排水干沟和重点湖泊污染治理，开展水环境联防联治，不断改善水环境质量。抓好贺兰山自然保护区清理整治，加强矿区生态环境恢复治理，推进固体废弃物资源化、减量化、无害化处理，治理农业面源和畜禽养殖污染，着力净化土壤环境。加强环境执法监管，严查环境违法行为。

强力推进节能降耗。坚持走循环发展之路，加快国家循环经济示范城市建设，完成石嘴山经开区、生态经济开发区循环化改造。支持企业开展烟气余热综合利用，构建循环经济产业链，培育30家循环经济示范企业，推进企业间、行业间、产业间共生耦合。实施重点用能企业能效提升计划，深入推进资源综合利用“双百工程”，严格实行能源消耗、水资源消耗、建设用地等总量和强度双管控，高效利用能源资源，全面完成自治区下达的目标任务。

（六）着力推进共建共享，全面增进民生福祉。坚持发展为民、成果共享，不断增加优质公共服务供给，守住底线、补齐短板，努力让人民生活更有保障，城市更加包容，社会更加温暖。

促进就业和居民增收。健全城乡统一的就业服务体系，统筹做好城市下岗职工、高校毕业生、农村转移劳动力、贫困人群等重点群体就业工作。加强技能培训，以创业带动就业，实现更加充分、更高质量的就业。实施居民增收计划，落实帮扶解困措施，提高城镇困难群体收入，多渠道增加农民经营性、工资性和财产性收入，努力实现居民收入增长和经济发展同步，缩小城乡收入差距，促进共同富裕。

坚决打赢脱贫攻坚战。全力实施脱贫攻坚行动计划，以产业带动为重点，健全完善就业创业、社会保障和对口帮扶等机制，引导贫困群众自力更生、艰苦奋斗，靠辛勤劳动实现脱贫致富。按照“三年集中攻坚、两年巩固提高、力争提前脱贫”的目标要求，实行返贫“挂号”、脱贫“销号”动态管理，确保全市3个贫困村全部摘帽，建档立卡贫困人口全部脱贫。

织密织牢社会保障网。健全完善各项社会保险制度，不断扩大城乡居民基本养老保险、医疗保险和大病保险覆盖范围。完成机关事业单位养老保险制度改革，优化“五险合一”经办机制，拓展社会保障“一卡通”功能，加快推进异地就医直接结算。完善城乡居民最低生活保障和社会救助制度，提高救助标准，扩大救助范围。健全养老设施和服务网络，大力发展社区养老、居家养老。切实保障妇女儿童权益，积极支持慈善事业、老龄事业、残疾人事业发展。

加快发展教育事业。坚持教育优先发展，全面实施优质教育资源扩面工程和质量提升计划，优化教育资源配置，加大政府购买学前教育服务力度，巩固义务教育均衡发展成果，推动高中阶段教育多元化发展，助推宁夏理工学院由普通本科高校向应用型高校转变。加大教育信息化建设力度，充分发挥信息技术在教育改革和质量提升方面的支撑引领作用，促进优质教育资源共享。

提高人民健康水平。实施健康促进行动，大力发展体育事业和体育产业，完善城乡体育设施，广泛开展全民健身运动；加强公共卫生和重大疾病防控，推进医疗养老结合，提高市民健康素养，争创全国健康城市。深化医药卫生体制改革，全面推进公立医院综合改革，实行医疗、医保、医药“三医”联动，建立现代医院管理、人事薪酬等制度，实施分级诊疗，促进优势资源下沉。加强医疗卫生机构重点学科建设，提升城乡医疗卫生水平。落实全面两孩政策，促进人口均衡发展。

繁荣发展文化事业。大力实施文化惠民工程，巩固提升国家公共文化服务体系示范区创建成果，加大政府购买公共文化服务力度，扩大文化资源向市民免费开放范围，引导文化资源向城乡基层倾斜，培育一批具有时代特征、石嘴山特色的文化精品，丰富广大群众的精神文化生活。加强文物保护和非物质文化遗产挖掘、整理、申报工作。积极培育新兴文化业态，提升文化产业消费水平。

（七）着力推进治理创新，全面加强社会建设。坚持治理与服务相结合，创新社会治理体制，改进社会治理方式，提升社会治理水平，不断提高群众幸福感和满意率。

加快推进依法治市。深入推进依法行政，建设法治政府，切实提高各级政府及部门的法治能力和水平。加强政府立法工作，提高立法质量，使立法反映群众意愿、适应发展需要。支持司法机关依法独立行使职权，保障广大群众有序参与司法活动。完善行政执法综合联动机制，加大重点领域执法力度，促进严格规范公正文明执法。认真落实“七五”普法规划，深入开展法治宣传教育，提高依法治市水平，创建全国法治先进城市。

健全公共安全体系。深化“平安石嘴山”建设，完善立体化社会治安防控体系，打好禁毒人民战争，依法打击违法犯罪活动。牢固树立安全发展理念，严格落实安全生产责任制，按照“党政同责、一岗双责、齐抓共管、失职追责”的要求，强化重点行业、重点领域安全隐患排查和专项治理，坚决遏制重特大事故发生，确保人民群众生命财产安全。加强应急体系建设，提高突发事件应急处置能力。加强农产品、食品质量安全监管，全面提高食品安全保障水平，争创国家食品安全示范城市。

提升社会治理能力。优化城乡社区网格化服务管理，深化和谐社区创建，完善社会矛盾排查预警和调处化解机制，推动“大调解”体系向纵深延伸，提升社会服务管理精细化水平。畅通和规范群众诉求表达渠道，落实领导干部接访、下访、包案化解信访问题机制，大力推进阳光信访、法治信访。鼓励和引导社会组织参与社会事务管理，增强基层社会自治能力。持续深入开展民族团结进步创建活动，提高宗教工作法治化水平，努力争创全国民族团结进步示范市。扎实做好新形势下双拥工作，加强国防教育，推动军民融合深度发展，不断巩固和发展民族团结、宗教和顺、社会和谐稳定的良好局面。

各位代表，决胜全面小康社会，是全市人民的共同期盼。我们坚信，只要咬定目标不放松，心无旁骛干事业，走新路，创新业，一个经济繁荣、民族团结、环境优美、人民富裕的石嘴山必将展现在我们面前！

三、2017年重点工作

2017年，是新一届政府的开局之年，也是实施“十三五”规划的关键一年。做好明年政府工作，要按照中央、自治区的总体部署和市第十次党代会要求，统筹抓好稳增长、促改革、调结构、惠民生、防风险各项工作，努力保持经济社会平稳健康发展。主要预期目标是：地区生产总值增长7%左右；固定资产投资增长8%左右；一般公共预算收入增长3%；城乡居民人均可支配收入分别增长7.5%和8.5%；城镇登记失业率控制在4.5%以内；完成自治区下达的各项约束性指标。

（一）切实抓好重大项目建设。强化项目建设对发展的支撑引领作用，实施重点项目110个以上，完成年度投资240亿元以上。重点抓好国电大武口热电联产、大唐火电、格林兰德环保等55个产业项目，完成投资160亿元以上；抓好红崖子黄河公路大桥、京藏高速改扩建石嘴山段、石银高速石嘴山至平罗联络线等35个基础设施项目，完成投资55亿元以上；抓好棚户区改造、北武当河生态治理（二期）等20个民生和社会事业项目，完成投资25亿元以上。紧盯国家和自治区重大战略、重大工程、重大活动，谋划储备项目600亿元以上。完善招商引资考核办法，抢抓全国工商联常委会暨民营企业助推宁夏创新发展大会召开机遇，大力开展专项招商，确保完成招商引资900亿元以上，努力增加有效投资。加大项目建设融资力度，争取中央专项建设基金10亿元以上，优化投资结构，放大投资效应，撬动社会资本投入。

（二）切实抓好工业稳定增长。坚持以提高质量和核心竞争力为中心，推动工业企业实施新一轮技术改造，重点抓好日盛化工、晟晏实业循环经济等20个技改升级项目建设，着力延伸和完善产业链条；开工建设北玻硅巢新材料、杉杉能源二期、国晶科技蓝宝石加工等18个调结构、增后劲的项目；加快推进大地轮胎、吉元冶金矿物棉、大窑饮品等15个扩规模、增总量的项目建设，确保年内建成投产。加强和改进服务企业工作，完善市级领导包抓重点企业、包推重点项目等工作机制，抓好50个工业增长点，新增产值50亿元以上。认真落实中央、自治区各项惠企政策，及时出台支持实体经济发展政策措施，帮助企业开拓市场、恢复生产。严格执行环保、能耗、质量、安全等相关法律法规和标准，创造条件推动企业兼并重组。加大中小微企业培育和拟入规企业扶持力度，力争12家企业进规入库，不断扩大工业经济总量，实现规上工业增加值增长7%以上。

（三）切实抓好现代服务业发展。打造科技金融广场，引进建设互联网金融服务平台、金融资产交易中心、大宗商品交易中心等金融机构。搭建融资平台，为开发区和企业提供融资服务。完成《大沙湖旅游区发展总体规划》编制，实施旅游交通通达、西线景观廊道工程，把沙湖作为宁夏旅游品牌向北延伸，加快北武当景区、奇石山文化旅游区、煤城·记忆等景区景点建设，推动全域旅游发展，提升旅游消费水平。加快推进中阿物流园平罗、惠农铁路物流中心建设，高标准建设石嘴山保税物流中心（B型），带动现代物流业发展。突出抓好消费层次提升，力促万达广场商贸综合体投入运营。建设乐天电商创业园、特色食品电商创业园，促进电子商务加快发展。力争服务业增加值增长8%左右。

（四）切实抓好农业提质增效。深入推进农业供给侧结构性改革，狠抓农产品标准化生产、品牌创建、质量安全监管。加快国家农业科技园区、河东现代农业示范区、贺兰山东麓休闲生态农业观光带建设，提升示范带动作用；建设30个优质粮食、草畜、水产、制种、瓜菜示范园区，培育新型农业经营主体20个，推动“一优五特”产业稳步发展；引导农民适应市场需求调整种植结构，推广节本增效农业新技术，加强肉羊、肉牛新品种繁育及推广体系建设，助推农民增收。继续抓好以平罗农村综合改革试点为重点的农村改革，释放农村发展活力。加大农业基础设施投入力度，建设高标准农田6.2万亩，改造中低产田10万亩，新增节水灌溉14万亩，提高农业综合生产能力。

（五）切实抓好城乡统筹发展。扎实开展空间规划（多规合一）改革试点，科学编制全市空间规划，提高空间管控水平和治理能力。深入推进“六城联创”，引导企业、社会组织和广大市民投身创建活动，实现城市共治共管、共建共享。建成智慧城市管理平台，启动智慧小区建设试点。开展环星海湖、城市主要出入口和主干道路综合整治，着力改善城市环境面貌。推进城市地下综合管廊建设，建成投入运营4.7公里，新开工建设10.8公里；新建改建静安街等城市道路5.6公里，完成城市零散棚户区改造638户。落实棚户区改造货币化安置政策，建立租购并举的住房制度，化解房地产库存，促进房地产市场平稳健康发展。建设特色小镇1个、美丽小城镇2个、美丽村庄8个，改造农村危房200户。

（六）切实抓好生态环境改善。全力做好中央环保督察组反馈的32个问题整改落实，努力解决环保突出问题。严格自备电厂能耗指标考核，抓好国电大武口电厂、石嘴山电厂火电机组超低排放改造，全面完成20蒸吨以上燃煤锅炉综合治理和城区及周边燃煤小锅炉淘汰。加强水污染防治，监督企业污水达标排放，推进城市污水厂提标改造；实施沙湖、星海湖水系连通工程，开挖连通水道6.2公里，对新开挖水道两侧水生态进行修复；切实做好三排、五排和沙湖、星海湖水污染治理；加强饮用水水源地保护，严格地下水超采区治理，关闭自备井12眼。加快贺兰山自然保护区清理整治，争取保护区规划调整。抓好市民休闲森林公园、归韭沟生态公园、城市绿地改造提升等11个生态项目建设，完成造林面积2.5万亩。

（七）切实抓好开发区环境提升。对标宁东能源化工基地，从整体形象、基础设施配套、产业承载能力等方面，全力推进开发区提档升级。实施电网优化、土地整理、生态绿化和废弃老旧厂房清理整治4个专项行动，抓好环境综合整治，切实改善开发区发展环境。完善开发区基础设施，新建园区道路51公里，建成运营石嘴山经开区、宁夏精细化工基地污水厂，开工建设石嘴山生态经济开发区污水厂及集污管网。完成正义关固体废弃物填埋场和综合利用项目建设，实现工业固体废弃物集中处理。推进园区循环化改造，切实降低企业生产要素成本，打造低成本园区，提升开发区综合竞争力。

（八）切实抓好抓实社会治理。建设12345服务平台，整合各职能部门服务热线，集中受理和办理市民咨询、求助、投诉事项。推进市、县区综治中心规范化建设、信息化管理，完善乡镇（街道）综治中心、民生服务中心功能，推进社区减负增效，夯实基层基础工作。建立城乡社区协商制度，每个县区培育3-5个城乡社区协商示范点，保障居民依法合理表达利益诉求。加强依法治理，多元化解社会矛盾纠纷，解决一批信访突出问题。启动实施“雪亮工程”，加强社会治安管理，坚决打击毒品违法犯罪活动。扎实推进社会信用体系建设，着力优化发展环境。深刻汲取林利煤矿“9.27”瓦斯爆炸事故惨痛教训，全力抓好安全生产工作，推动安全生产形势稳定向好。

（九）切实抓好民生改善工作。优化财政支出结构，继续向民生领域和薄弱环节倾斜。加快推进医药卫生体制、公立医院综合改革，更好的满足群众健康需求。完成星海中学、青少年示范性综合实践基地、妇幼保健院等项目建设，力争年底前投入使用。深化现代公共文化服务体系建设，实施国家艺术基金、明长城维修保护项目。开工建设全民健身中心。积极做好城镇职工医疗保险自治区级统筹工作。扎实推进救助制度与扶贫开发政策有效衔接，切实做好脱贫攻坚和帮扶解困工作。

本着群众关切、量力而行的原则，继续办好一批民生实事。1.完成老旧住宅小区节能改造50万平方米以上；2.改造城市旱厕50座以上；3.完成50个老旧住宅小区技防改造项目；4.对城乡贫困妇女进行“两癌”免费筛查；5.建设石嘴山市数字文化馆；6.开展关爱困境妇女儿童“五个五”项目行动；7.在三县区各建设一个国民体质监测中心；8.实现农村80周岁以上老年人高龄津贴城乡一体化；9.开展城乡劳动力技能培训2000人、创业能力培训1000人；10.购置清扫车、喷雾车各10辆，用于城市区（开发区）道路清扫和喷雾降尘，改善环境质量。

四、加强政府自身建设

各位代表，新形势、新任务对政府工作提出了新的更高要求。我们要按照实干、高效、担当、清廉的要求，恪守为民之责，勇担发展重任，切实提高施政能力和服务水平，努力建设人民满意的政府。

（一）脚踏实地，真抓实干。为政之要，重在实干。坚持“干”字当头、“实”字为要，把全部心思和精力用到干事创业上，以踏石留印、抓铁有痕的精神，抓落实、办实事、促发展。坚持依法决策、依法行政、依法履职，建立完善政府与市人大代表联系制度，加大政务公开力度，主动接受人大及其常委会法律监督、政协民主监督和社会各界监督。对中央和自治区党委、政府的决策部署坚决执行，对市委、政府定下来的事情一抓到底，对重大事项和关键环节紧盯不放，高标准、高质量完成各项工作。始终牢记和践行为人民服务宗旨，善谋实事、多做实功，注重打基础、谋长远，不急功近利，不做表面文章，不折不扣落实国家、自治区出台的各项惠民政策，说到的就要做到，承诺的就要兑现。

（二）执行有力，务实高效。执行力是政府工作的生命力。强化时间观念、效率意识，大力倡导立说立行、雷厉风行的工作作风，接受任务不讲条件，执行任务不找借口，坚决杜绝议而不决、决而不行、行而不果现象，切实提高决策效率，提升办事速度，推动工作高效运转，把决策和思路真正变为现实。完善部门季度考评、电视问政、干事档案等监督考评机制，健全目标管理机制，对重点项目、重大事项、重要工作实行项目化清单管理，以工作目标倒逼实施进度，层层传导压力，逐级压实责任，确保事事有着落、件件有结果。健全完善督查问效机制，实行进度通报、对账销号，强化绩效考核，严格兑现奖惩，确保政令畅通、执行有力。

（三）主动作为，敢于担当。重任千钧惟担当。面对严峻复杂的经济形势、艰巨繁重的发展任务，敢于担当，敢于负责，特别是在矛盾和问题面前敢抓敢管、敢于碰硬，在急难险重任务面前迎难而上、勇挑重担，不回避、不推诿、不退缩、不懈怠，真正肩负起时代和人民赋予的责任和使命。强化争先创优意识，争先进位、比学赶超，千方百计争政策、争项目、争资金。建立健全鼓励担当的容错机制和惩治庸政、懒政、怠政行为的问责机制，鼓励探索，支持创新，宽容失误，对真抓实干成效明显的县区和部门给予鼓励和政策倾斜，为干事创业的人担当，为敢于担当的人担当，努力消除不作为、乱作为、慢作为现象，促进全市各级干部奋发有为、干事创业。

（四）严以用权，清正廉洁。为政清廉才能取信于民，秉公用权才能赢得人心。坚持把纪律和规矩挺在前面，不断增强政治意识、大局意识、核心意识、看齐意识，严格执行党风廉洁建设各项规定，扎实推进政府系统反腐倡廉工作，自觉做到按制度办事、按程序办事、按规矩办事。健全权力运行制约和监督机制，充分发挥行政监察、审计监督作用，加强对重点部门、关键岗位的监管，切实把权力关进制度的笼子里。强化过“紧日子”的意识，严格预算管理，厉行勤俭节约，降低行政成本。坚决落实中央“八项规定”精神，坚持不懈纠正“四风”，严肃查处违纪违法行为，营造风清气正的政务环境，树立“为民、务实、清廉”的政府形象。

各位代表，实干才能实现梦想，奋斗才能赢得未来。让我们更加紧密地团结在以习近平同志为核心的党中央周围，在自治区党委、政府和市委的坚强领导下，大力弘扬“不到长城非好汉”的宁夏精神和“五湖四海、自强不息”的石嘴山精神，以昂扬进取的精神状态、只争朝夕的豪迈干劲，转型发展走新路、全面振兴创新业，全力推进产业民生生态转型，加快建设开放富裕和谐美丽石嘴山，为决胜全面小康而努力奋斗！
